# Development of Histamine in Fresh and Canned Tuna Steaks Stored under Different Experimental Temperature Conditions

**DOI:** 10.3390/foods11244034

**Published:** 2022-12-14

**Authors:** Alberto Altafini, Paola Roncada, Alessandro Guerrini, Gaetan Minkoumba Sonfack, Damiano Accurso, Elisabetta Caprai

**Affiliations:** 1 Department of Veterinary Medical Sciences, Alma Mater Studiorum—University of Bologna, Via Tolara di Sopra 50, 40064 Ozzano dell’Emilia, Italy; 2 Department of Environmental Science and Policy, University of Milan, Via Celoria 10, 20100 Milan, Italy; 3 Reparto Chimico Degli Alimenti, Istituto Zooprofilattico Sperimentale della Lombardia e dell’Emilia Romagna “Bruno Ubertini”, Via P. Fiorini 5, 40127 Bologna, Italy

**Keywords:** biogenic amines, food safety, food storage, foodborne intoxication, histamine, histidine decarboxylase, LC-MS/MS, tuna

## Abstract

Among biogenic amines, histamine is most frequently involved in foodborne intoxication. To evaluate histamine formation in tuna, several storage conditions were reproduced. An LC-MS/MS method was used for analytical determinations. Fresh tuna samples (not contaminated and grafted with tuna muscle naturally incurred with histamine at 6000 mg/kg) were stored at 4, 12, and 20 °C, and daily samples were collected for 6 days. The development of histamine was observed only in grafted tuna samples. At 4 °C, histamine formation progressed from 12.8 mg/kg (day 1) up to 68.2 mg/kg (day 6). At 12 °C, higher concentrations developed (23.9 mg/kg on day 1 up to 2721.3 mg/kg on day 6) relative to 20 °C (from 12.0 to 1681.0 mg/kg). It was found that at 4 °C, if grafted tuna was submerged in oil, histamine formation progressed more slowly. In a naturally contaminated sample, it was observed that the histamine distribution was uniform, while the normal cooking process did not affect the histamine level. Furthermore, it was found that the use of histamine-contaminated equipment for food handling may result in histamine formation in food. These results confirm the importance of implementing good hygiene practices and respecting the cold chain.

## 1. Introduction

The name “tuna” covers 14 species belonging to 4 different genera (*Auxis*, *Katsuwonus*, *Euthynnus*, and *Thunnus*), which can be found in almost all seas of the world. This large family of fish is of major economic importance in a fully globalized economy [1]. Many countries are dependent on tuna resources for food supply and nutrition, economic development, employment, culture, and recreation. On a global level, we are witnessing the increasing consumption of canned tuna, as it is practical, versatile in its preparation, and rich in nutritional properties. The whole tuna market size reached a value of USD 57.96 billion in 2021. The market is further expected to grow at a CAGR (Compound Annual Growth Rate) of about 5.1% in the forecast period between 2022 and 2027 to reach a value of about USD 78.20 billion by 2027 [2]. European countries and the U.S. are the largest consumers of canned tuna. Thailand, Ecuador, China, Indonesia, and the Philippines are the largest exporters of canned and processed tuna, while the U.S., Japan, Australia, Egypt, Saudi Arabia, and European countries are the largest importers of these products [3]. According to 2020 data, the Italian market is worth more than EUR 1.40 billion, with a national production of over 80,300 tons and a consumption of over 160,000 tons (about 2.67 kg per capita). A comparison with previous years shows exponential and constant growth in Italy and abroad, EU and non-EU. Compared to 2011, the national market value of canned tuna has increased by 31.3%, Italian production has increased by 18%, per capita consumption has increased by 17.4%, and imports have increased by 12.5%. These data confirm Italy as one of the most important markets in the world for the consumption of this food and as the second largest European producer, after Spain [4]. The COVID-19 pandemic crisis has also increased this market trend. Travel restrictions and the consequent need to stock up on food have influenced the eating habits of Italians, especially during the lockdown phase. In particular, in the initial weeks of the emergency, the consumption of canned tuna increased by 33.6%, while in the first 5 months of 2020, volume sales stood at 33,810 tons. More than one in three Italians (36%) said they consume it more than 2–3 times a week due to its long shelf-life, versatility, and flavor [5]. 

Unlike canned tuna, fresh tuna is a high-value product, with demand mostly concentrated in Southern Europe (Spain, France, and Italy). This product is mostly consumed in the food service sector and can be purchased in specialized retail stores in fresh or refreshed (defrosted) forms [6]. Among the food trends of the last 10 years, there has been a growing popularity of sushi and sake that rely on fresh tuna. In addition, there are now more and more restaurants that offer dishes such as tartar or grilled tuna. These trends are factors that have collectively contributed to the increased demand for this type of product [7]. Fresh tuna is a special product with particular requirements to maintain its color, freshness, and quality. It is usually distributed as loins under vacuum, from which steaks are made. Tuna loins are often packed in modified atmosphere packaging (MAP), which also ensures a longer shelf-life [6]. In most cases, fresh tuna sold in fish markets or served in restaurants is the yellow-fin tuna species (*Thunnus albacares*), which can come from the Atlantic, Indian, or Pacific Ocean. However, the most renowned species is blue-fin tuna (*Thunnus thynnus*), which is fished for in the Mediterranean Sea. Except for a very small part that is sold fresh, destined for fishmongers and upmarket restaurants, blue-fin tuna is sold alive to other countries, mainly to Spain, to be fattened inside huge floating cages and then to be resold all over the world (including Italy) [7].

Several food frauds involving this type of product have been reported, especially attempts to market less valuable species as blue-fin tuna or to sell a thawed product as fresh. Treatments of tuna with nitrites/nitrates and/or carbon monoxide to change the color of the fish muscle are practices often implemented by fraudulent fishery operators. These illegal treatments allow canning-grade tuna to be sold as sushi-grade tuna. It has been calculated that the profit from this type of illicit practice would be 8–10 EUR/kg, and considering the volumes involved, the economic gain could potentially be >EUR 200 million per year [8]. 

The Rapid Alert System for Food and Feed (RASFF), the notification system of the European Commission to exchange information on identified hazards between member states, records dozens of alerts related to tuna every year. Apart from those concerning the presence of mercury or other heavy metals, most of the noncompliances (about 80 from January 2020 to September 2021) are due to the presence of histamine and the interruption of the cold chain, but also due to the excessive use of permitted additives, the use of unauthorized additives such as carbon monoxide, or a lack of traceability [7]. Illicit treatment with additives can be hazardous to health. Artificially producing tuna’s bright red color may hide a product that is no longer fresh, without considering the potential hazard of the excessive intake of the additives themselves. In particular, nitrite in food (and nitrate converted to nitrite in the body) can oxidize hemoglobin to methemoglobin, an excess of which reduces the ability of red blood cells to bind and transport oxygen through the body. Furthermore, nitrite may also contribute to the formation of a group of compounds known as nitrosamines, some of which are carcinogenic [9].

It is essential for the consumer that foods preserve their organoleptic characteristics (taste, smell, appearance, and texture) and nutritional properties. Therefore, proper food storage is important since food components are susceptible to microbial, chemical, physical, and insect spoilage. More than 250 different foodborne diseases have been described. Most of these diseases are infections caused by a variety of bacteria, viruses, and parasites that can be found in food. In addition, poisonous chemicals or other harmful substances can cause foodborne diseases if they are present in food [10]. Certain foods are more vulnerable to biological contamination than others because they have certain characteristics that support the growth of pathogenic microorganisms. These characteristics include pH over 4.5, nutrients (high in starch or protein), and moisture. Examples of high-risk foods include meat and poultry (cooked or raw), eggs (cooked or raw), dairy products, seafood, prepared fruits and vegetables, unpasteurized juices, cooked rice, fresh or cooked pasta, and foods that contain any of the above [11]. 

The formation of biogenic amines (BAs) due to bacterial enzymatic activities in fish foods may be one of the consequences of improper storage and/or temperature abuse [12]. On the basis of the pathogenic mechanism, adverse reactions resulting from the ingestion of food can be classified into toxic and non-toxic reactions. Toxic reactions result from a primary harmful effect that the food has on all individuals who eat it [13]. This type of reaction mainly occurs after the ingestion of foods contaminated with infectious microorganisms (viral, bacterial, parasitic, or fungal) or toxins secreted during the different stages of food processing, production, or preservation [14]. Otherwise, non-toxic food reactions are caused by a particular individual intolerance toward commonly tolerated foods [13]. This last type of reaction is commonly distinguished into food allergies, due to an adverse immune-mediated response, and food intolerances that do not involve the immune system [15]; non-immune-mediated reactions are the most common [16,17].

Most food allergies are due to allergenic proteins and are IgE-mediated. Oral allergens can boost the production of allergen-specific IgE antibodies. The allergens cross-link the IgE on the surface of mast cells and basophils, which release certain inflammatory mediators into the surrounding microenvironment, particularly histamine, which is the main culprit in causing immediate allergic reactions [17].

Food intolerances are defined as reactions that are reproducible but non-immune-mediated, instead having enzymatic, pharmacological, or unknown causes [18]. Pharmacologic reactions to food are among the most frequent forms of food intolerance. They are defined as adverse reactions to foods that result from naturally derived or added chemicals (e.g., biogenic amines) that produce drug-like or pharmacological effects in the host [19].

Biogenic amines (BAs) are toxic nitrogenous compounds produced mainly by the microbial decarboxylation of their precursor amino acids [20]. Based on their chemical structure, BAs can be classified into three main categories: aliphatic amines comprising cadaverine and putrescine (diamines) and spermine and spermidine (polyamines); aromatic amines comprising tyramine and 2-phenylethylamine (monoamines); and finally, heterocyclic amines such as histamine [21,22,23]. These substances are not affected by the heat treatments to which foods may be subjected in the production process [24,25,26,27]. Moreover, BAs have been frequently considered markers of the freshness and hygiene of foodstuffs during storage [22,28,29,30]. Fish is the type of food on which the largest number of BA analysis studies have been carried out, and it is highly perishable. However, these substances can also be present in meat and meat products, wines of all kinds, cheeses, beers, various beverages and traditional sauces [28], dairy, fruits, vegetables, nuts, and chocolates [31]. High amounts of amines can be present in fermented foods derived from raw materials with high protein content, such as salami and dry sausages [32]. The most important biogenic amines found in foods are histamine, tyramine, putrescine, cadaverine, 2-phenylethylamine, spermine, spermidine, tryptamine, and agmatine. Furthermore, octopamine and dopamine have been detected in meat products and fish [29]. The main factors that affect BA formation are the presence of specific bacterial strains and the quality of the raw materials. Other conditions that need to be considered are temperature, pH, the availability of substrates (amino acids), water activity (Aw), NaCl content, redox potential [33], oxygen availability, certain additives, and competition among microorganisms [12].

Contrary to what might be expected, microbiological spoilage can also occur in salted products, since BA accumulation can occur before salting. Moreover, if sea or rock salt contains nitrate and nitrite as impurities, BAs in salted fish products may react with nitrites to form potentially carcinogenic nitrosamines [28]. The microbial agents responsible for the formation of biogenic amines in fish can be present in the bacterial flora of live fish, but most appear to result from contamination that occurs on board fishing vessels subsequent to capture, during processing in food plants, during distribution, or in restaurants or homes [34]. Tyramine and histamine are considered the most toxic BAs and are of the greatest relevance to food safety [35]. The consumption of foods rich in tyramine can cause toxicological reactions, together referred to as the “cheese reaction” because this BA is most frequently found in cheese [36]. The main symptoms are headaches, migraines, neurological disorders, nausea, vomiting, respiratory disorders, and hypertension [37]. Further signs associated with tyramine are the dilation of pupils and palpebral tissues, lacrimation, salivation, fever, and an increase in blood pressure [38].

Histamine is the type of amine most biologically important for its toxicological effects [39], in addition to being the most frequently involved in foodborne intoxication [30,40]. Histamine is also a naturally occurring substance in the human body. It is mainly produced by mast cells and basophils, where it is stored in granules. Once released as a result of various stimuli, both immunological and nonimmunological, it acts by binding to different types of histamine receptors on the surfaces of cells. It can be found in many tissues, including the skin, intestinal mucosa, heart, and lungs. This molecule is involved in many pathophysiological and physiological functions, such as gastric acid secretion, inflammation, and the regulation of vasodilation and bronchoconstriction. It can also be found in neurons of the central nervous system, where it acts as a neurotransmitter [41,42]. Histamine formed in food results from the decarboxylation of the natural amino acid histidine catalyzed by a specific enzyme, histidine decarboxylase (HDC), present in several kinds of bacteria [43]. Histamine, when taken in through food, is rapidly metabolized in the human gut by intestinal diamine oxidase (DAO) to less active products. In the case of the ingestion of foods with high histamine content or subjects with reduced DAO activity, the detoxification system is unable to eliminate histamine, which then binds to specific receptors, causing symptoms generally similar to those of IgE-mediated food allergies [44]. Clinical manifestations include cutaneous symptoms (skin rash, particularly localized on the face and neck, feeling of intense heat, urticaria, facial edema, welts, conjunctival hyperemia, and itching), gastrointestinal symptoms (diarrhea, abdominal pain, nausea, vomiting, burning, and swelling of the mouth and tongue), hemodynamic symptoms (hypotension and dizziness), and neurological symptoms (headaches, palpitations, tingling, vision disturbances, tremors, weakness, and feeling hot). Pungent or metallic taste sensations and oral insensitivity are also reported. Less frequent are signs involving the CNS, such as anxiety and excitation. In more severe cases, bronchospasm, respiratory distress, and cardiac complications are described, but these usually occur in individuals with pre-existing conditions [45,46,47,48]. In general, the onset of symptoms is rapid, ranging from immediate to 30 min after eating the implicated food [47,49]. However, in some cases, they can occur up to 60 min after food ingestion [50,51] or even up to 90 min after food ingestion [52,53]. Symptoms are generally fleeting and do not require treatment, resolving on their own within a few hours or at most within 24 h, but feelings of malaise may last for several days [45,47].

Histamine intoxication is commonly referred to as scombroid poisoning, but other terms that have been used for this intoxication are pseudoallergic fish poisoning, histamine overdose, or mahi-mahi flush [54]. The term “scombroid” is due to the fact that the fish species most often implicated belong to the families *Scombroidae* and *Scomberesocidae*, which include dark-fleshed fish such as mackerel, tuna, skipjack, bonito, and cero [46,55]. These species of fish share in common high levels of free histidine in their muscle tissue [56]. 

Scombroid poisoning is also classified as “allergy-like intoxication” because it can mimic the symptoms of food allergies, since histamine is their major chemical mediator. In fact, in true food allergies, histamine is released from intracellular locations, while in allergy-like intoxication, it is ingested with foods [57]. Currently, histamine is the only BA having official limits in fish products. In particular, at the European level, Commission Regulation (EC) No 2073/2005, as amended by Commission Regulation (EC) No 1441/2007 and Commission Regulation (EU) No 1019/2013, establishes that nine portions should be taken from each sample for histamine analysis, and the mean must not exceed 100 mg/kg; two samples may have a value of more than 100 mg/kg but less than 200 mg/kg, and no sample may have a value greater than or equal to 200 mg/kg [58,59,60]. In the United States, the Food and Drug Administration (FDA) has instead set a stricter limit for histamine (50 mg/kg) [61]. The determination of BAs in fresh and processed foods is thus of great interest not only due to their toxicity but also because they can be a useful index of spoilage or ripening [30]. The maintenance of controlled environmental conditions throughout the food chain is essential for the healthiness of the final product and, in certain types of food, for preventing conditions that allow the formation of BAs. Furthermore, the importance of the storage conditions of food between the time of purchase and the time of consumption should not be underestimated, especially if it is perishable food such as fish-based foods, and even more so if it is not consumed immediately after opening the package. The aim of this study was to reproduce in the laboratory some domestic storage conditions of food, tuna in particular, to evaluate the possibility of histamine development. For this purpose, tests were conducted on samples of tuna, both fresh and canned, also mixed with salad, and subjected or not to cooking, after which samples were kept under different temperature conditions for variable periods of up to 14 days. For analytical determinations, a liquid chromatography–tandem mass spectrometry system (LC-MS/MS) was used.

## 2. Materials and Methods

### 2.1. Samples

For this study, samples of fresh tuna (about 2 kg in total) and canned tuna from different brands, both in sunflower oil (4 packs of 160 g) and in water (2 packs of 80 g), were used. Fresh tuna was purchased at a fish market, while canned tuna was obtained in a supermarket, both located in the surrounding area of Bologna (Italy). Soon after purchase, the fresh tuna was stored at −20 °C, while the canned tuna was kept at room temperature. A portion was taken from each tuna sample to be analyzed for the presence of histamine. After opening, the canned tuna samples were also stored at −20 °C for further investigation. In accordance with the purposes of the present work, several trials were performed to evaluate the possible conditions for histamine development and, in particular, to evaluate histamine formation in fresh tuna steaks stored at different controlled temperatures (4 °C, 12 °C, and 20 °C) to determine the most favorable condition for the development of histamine; evaluate whether other foods mixed with tuna (e.g., vegetables) may carry histaminogenic bacterial strains; evaluate whether the equipment used to handle the food may be a vehicle for histamine contamination; assess whether the cooking process can reduce the histamine level of contaminated tuna preparations; and evaluate the distribution of histamine within a tuna steak. Some of these trials were performed using tuna steak samples naturally contaminated with histamine at the 6000 or 8000 mg/kg level, collected in the course of previous checks carried out on tuna samples involved in scombrotoxic poisoning incidents.

### 2.2. Trials

#### 2.2.1. Trial 1

Nine hundred grams of fresh homogenized tuna was divided into 3 aliquots, each placed in an aluminum tray and subjected to a different storage temperature (4, 12, and 20 °C, respectively). Another 900 g of the same tuna was instead grafted with 1 g of tuna muscle naturally incurred with histamine (approximately 6000 mg/kg) and placed under the same temperature conditions. Daily samples were taken from the different aliquots subjected to the experimental temperatures for 6 days (in total, 36 samples collected).

#### 2.2.2. Trial 2

A portion of fresh tuna grafted with 1 g of tuna muscle naturally incurred with histamine (approximately 6000 mg/kg) was placed in a beaker, submerged in corn seed oil, and stored at 4 °C. An aliquot of the sample was then taken every 2 days for 14 days (7 samples in total) and stored at −20 °C until analysis. The purpose of this test was to evaluate histamine formation under anaerobic conditions compared with the amount of histamine formed under aerobic conditions in the previous experiment.

#### 2.2.3. Trial 3

Four 160 g packs of canned tuna in sunflower oil were homogenized with about 140 g of canned corn, carrots, and three different types of lettuce (Gentile lettuce, red radicchio, and Trocadero lettuce), purchased in the fruit and vegetable department of a large retail supermarket. The prepared tuna salad was covered with aluminum foil and stored at 4 °C to assess whether the co-presence of other foods could be a vehicle for histaminogenic bacterial strains. Two aliquots of about 20 g of tuna were taken from the salad every day for 8 days for a total of 16 samples. Special care was taken in daily sampling, which was carried out at different points and after thoroughly mixing the tuna preparation.

#### 2.2.4. Trial 4

A tuna steak that tested positive for histamine (mean concentration of 8000 mg/kg) was used to assess whether the contamination of the steak was homogeneous. For this purpose, samples were taken from both ends and the core of the product.

#### 2.2.5. Trial 5

From a tuna steak that tested positive for histamine (average concentration of 8000 mg/kg), 3 slices were taken and then subjected to cooking in order to assess how much this process could reduce the level of contamination.

#### 2.2.6. Trial 6

The cutting board employed to portion a tuna steak that tested positive for histamine (concentration level of 8000 mg/kg) was used to check for possible contamination of other food products via contact. For this aim, 4 slices of uncontaminated fresh tuna were placed on the cutting board at room temperature. The slices were then cut into small pieces to increase contact with histamine and/or histaminogenic bacterial strains that may have been present on the surface of the cutting board. An aliquot was taken from each tuna slice to assess for the presence of histamine and to check whether the contamination was homogeneous. The same experiment was performed using moist salad, which was rubbed and cut on the cutting board and subsequently placed in contact with 160 g of uncontaminated tuna in water. An aliquot of about 20 g of this tuna was then sampled to assess for the presence of histamine.

### 2.3. Solvents and Reagents

Methanol (LC-MS grade) and ammonium acetate (analytical grade) were purchased from VWR Chemicals (Milano, Italy). Potassium dihydrogen phosphate (analytical grade) and acetonitrile (LC-MS grade) were from Sigma-Aldrich (St Louis, MO, USA). Formic acid (analytical grade) and hydrochloric acid 1N were from Carlo Erba Reagents (Cornaredo, MI, Italy). Ultrapure water used throughout the experiments was produced by an Evoqua Water Technologies system (Pittsburgh, PA, USA). Histamine dihydrochloride (≥99.0%), used as a reference standard, was supplied by Sigma-Aldrich Co. (St Louis, MO, USA).

### 2.4. Chromatographic Apparatus and Conditions

The LC-MS/MS system used was an Acquity ultra-performance liquid chromatograph (UPLC) (Waters, Milford, MA, USA) coupled to a Quattro Ultima Platinum triple-quadrupole mass spectrometer with an electrospray ionization source (Micromass, Manchester, UK). The system was computer-controlled, and data acquisition, peak integration, and calibration were performed using Mass Lynx software (Micromass, Manchester, UK). LC separation was achieved in gradient elution mode and at room temperature by means of an analytical column (Luna HILIC, 3 µm 150 × 2 mm; Phenomenex, Torrance, CA, USA). The mobile phase consisted of 15 mM ammonium formiate in water (A) and methanol (B). The best conditions for the analyte separation were obtained with isocratic elution with 80% A and 20% B for 5 min. The flow rate was set at 0.25 mL/min, and the injection volume was 10 µL. The MS parameters were optimized in positive ionization mode (ESI+). The capillary voltage, cone voltage, source temperature, and desolvation temperature were set at 3.5 kV, 40 V, 120 °C, and 450 °C, respectively. The direct infusion of a pure histamine standard solution was used to identify the molecular ion (M + H+), followed by a product ion scan to identify the most prominent fragments following collision energy optimization. This information was used to determine the appropriate selected reaction-monitoring (SRM) transitions that were used during the analysis. The ion transitions and mass parameters monitored for histamine are reported in Table 1.

### 2.5. Sample Preparation

The extraction procedure was carried out following the method described by Altafini et al. [62] with slight modifications. Aliquots of 1 ± 0.1 g of chopped tuna samples were weighed into a 50 mL Falcon tube. After the addition of 20 mL of 50 mM methanol–phosphate buffer (50:50, *v*/*v*), each sample was mixed on a horizontal shaker for 30 min and centrifuged at 10,000 rpm in a refrigerated centrifuge at 5 °C for 10 min. The resulting extraction solution was then diluted 1:100 (*v*/*v*) with 0.1 N HCl, and 10 µL was injected into the LC-MS/MS apparatus for analysis.

### 2.6. Quantification

A series of histamine standard solutions in the solvent at different concentrations were prepared to create calibration curves. Each standard solution was analyzed by LC-MS/MS, and, using the data system software, the analytical response (peak area) was plotted against the concentration in a system of Cartesian axes to obtain a calibration curve. The plot was linear, and the regression equation was used to calculate the concentration values of histamine in unknown analytical samples from the peak areas resulting from LC analysis. These values were then multiplied by the dilution factor (1:2000) resulting from the sample preparation procedure to obtain the histamine concentrations in the matrix.

## 3. Results

### 3.1. Method Performance

**Figure 1:**
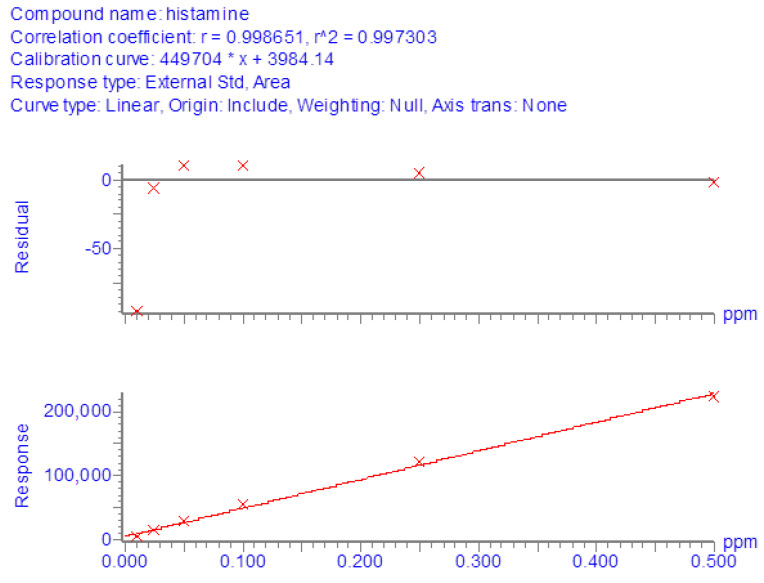
Calibration curve in solvent generated from the analysis of pure histamine standard solutions at concentrations from 10 to 500 ng/mL.

For LC-MS/MS determinations, reference curves in the solvent were generated from the analysis of pure histamine standard solutions at concentrations from 10 to 500 ng/mL. All regressions showed satisfactory linearity since the coefficient of determination (R^2^) was always greater than 0.99 (Figure 1). 

**Figure 2:**
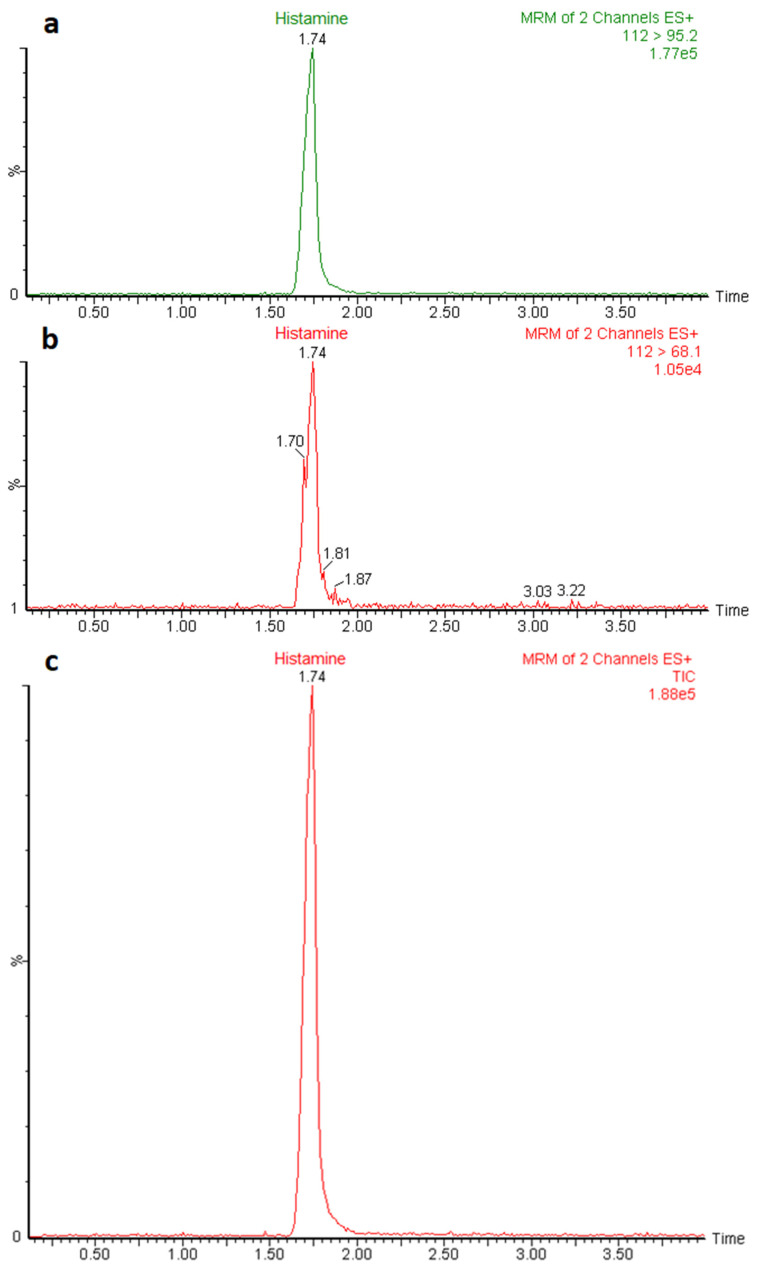
Chromatograms obtained after LC-MS/MS analysis of a histamine standard solution at 25 ng/mL level representing the quantitation (*m*/*z* 112 > 95.2) (**a**) and confirmation (*m*/*z* 112 > 68.1) (**b**) MRM transitions, and total ion current (TIC) chromatogram (**c**).

Possible matrix-induced interferences were evaluated by analyzing 20 blank samples from different types of fish and spiked samples. No significant peaks were found within the retention time window of histamine in the non-contaminated samples, and no interfering peaks were observed in the spiked samples. The retention time of histamine was about 1.75 min, while the total run time was 5 min (Figure 2).

Recovery experiments were carried out at three spike levels (20, 100, and 200 μg/g) by comparing the peak area of histamine in spiked samples and the peak area of histamine in pure standard solutions at the same concentration levels. The average recovery percentages calculated from 18 replicates for each concentration were found to be between 78.8% and 82.9%, while the overall average recovery was 80.8% (Table 2).

For each analytical batch, quality control checks were performed by fortifying a matrix blank at the 100 mg/kg level and analyzed together with the unknown samples. To evaluate the repeatability of the analytical method, intraday tests were performed. Analyses were carried out with the same instruments, on the same day, and by the same operators. Six spiked samples at three concentration levels (20, 100, and 200 μg/g) were prepared and analyzed (eighteen determinations in total). Similarly, the within-laboratory reproducibility was evaluated on 6 blank samples fortified at 20, 100, and 200 mg/kg, but in this case, the procedure was carried out on 3 different days and by different operators (54 determinations in total). The RSDs of the quantification results ranged from 8.8 to 9.6% and from 8.8 to 13.1% for repeatability and within-laboratory reproducibility, respectively (Table 3).

The limit of detection (LOD) of the analytical method was 1 mg/kg, while the limit of quantification (LOQ) was 20 mg/kg. LOD was calculated on the basis of a signal-to-noise ratio (S/N) of 3:1, while LOQ was the concentration of histamine in the matrix corresponding to the lowest point of the calibration curve. These results were satisfactory for the purposes of the study, considering that LOD and LOQ were well below the histamine limit (100 mg/kg) established at the EU level.

### 3.2. Occurrence of Histamine in Tuna Samples Stored under Different Experimental Conditions

#### 3.2.1. Trial 1

In this experiment, a portion of fresh tuna not contaminated with histamine was divided into three aliquots, each stored at a different temperature. To assess for histamine formation, a daily sample was taken from each aliquot on six different days, but no sample was found to be contaminated. On the last day, tuna samples stored at 12 and 20 °C appeared markedly deteriorated and moldy due to unrefrigerated storage. However, histamine was also not detected in these samples (Table 4). This suggests that the spoilage of food with a high amount of histidine does not necessarily lead to histamine formation, but the presence of specific histidine-decarboxylating bacteria (HDB) is necessary. In contrast, the sampling of the other portion of the same tuna that was grafted with histamine-contaminated tuna at a concentration of 6000 mg/kg and stored at the same storage temperatures showed quite different results (Table 4).

**Figure 3:**
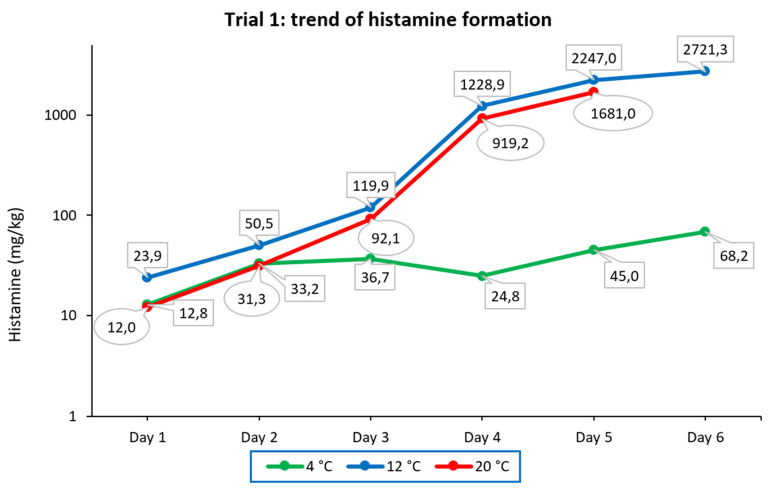
Trend of histamine formation over six consecutive days in fresh tuna samples grafted with tuna muscle naturally incurred with histamine and stored under different temperature conditions (trial 1). The ordinate is scaled logarithmically.

As shown in Figure 3, in samples stored at 4 °C, histamine formation progressed rather slowly, from 12.8 mg/kg detected on day 1 to 68.2 mg/kg detected on day 6. At 12 °C, the histamine concentration detected on day 1 was 23.9 mg/kg, and it grew exponentially on day 2 and day 3, with the largest increase occurring on day 4, as it was increased 10-fold. Measurements taken on day 5 and day 6 showed a decrease in histamine growth, which was increased 1.8- and 1.2-fold, respectively. Finally, in samples stored at 20 °C, the concentrations detected were lower than those measured in samples stored at 12 °C. However, the trends of histamine formation under these two storage conditions were quite similar. The daily increases in histamine from day 2 to day 5 were 2.6-, 2.9-, 10-, and 1.8-fold, respectively. On the last day, the tuna sample became completely moldy, and it could not be analyzed.

#### 3.2.2. Trial 2

**Figure 4:**
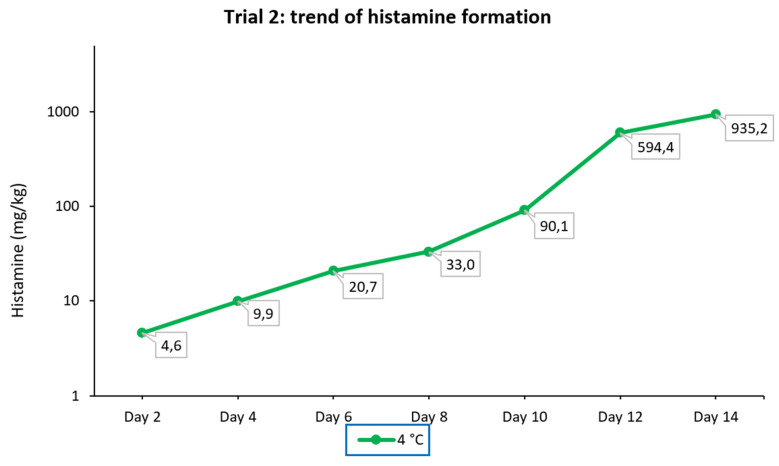
Trend of histamine formation over fourteen consecutive days in fresh tuna samples grafted with tuna muscle naturally incurred with histamine, submerged in corn seed oil (anaerobic condition), and stored at 4 °C (trial 2). The ordinate is scaled logarithmically.

In this experiment, the sampling of the portion of fresh tuna submerged in corn seed oil (anaerobic conditions) and stored at 4 °C for 14 days showed lower levels of histamine in the first 6 days compared with those detected in trial 1 in tuna samples stored at the same temperature but under aerobic conditions. From day 8 to day 10, the amount detected tripled, while subsequent sampling showed a 6.7-fold growth in the histamine level between day 10 and day 12, followed by a slowdown in growth (1.6-fold) in the last 2 days. The concentrations detected in this experiment and the trend of histamine formation are displayed graphically in Figure 4.

#### 3.2.3. Trial 3

Daily sampling followed by a laboratory analysis of canned tuna in sunflower oil mixed with vegetables showed that the latter were not vehicles for histamine-producing bacteria. In fact, none of the 16 samples collected during the 8-day trial in which the tuna was stored at 4 °C showed traces of histamine.

#### 3.2.4. Trial 4

This experiment showed that in the contaminated tuna sample analyzed, histamine was fairly evenly distributed, as the average concentrations detected on the outer surface and inner part of the product were very similar (10,135.1 and 11,864.6 mg/kg, respectively). 

#### 3.2.5. Trial 5

Measurements made on the histamine-contaminated tuna steak subjected to the normal cooking process showed that the concentration of histamine was almost unchanged from that assessed before cooking (9400.9 and 9327.1 mg/kg, respectively).

#### 3.2.6. Trial 6

The purpose was to assess whether equipment under unhygienic conditions used to handle food can potentially be a vehicle for histamine contamination. The test showed the uneven histamine contamination of tuna due to contact with the histamine-contaminated cutting board in both fresh tuna and tuna salad, as reported in Table 5.

## 4. Discussion

The experiments carried out in the present study showed that the presence of HDB is essential for histamine formation, and the temperature factor alone is not sufficient to trigger this process. In fact, in the experiment conducted at room temperature, after 5 days, the tuna showed marked spoilage signs and changes in organoleptic characteristics due to improper storage, but no histamine formation was detected. The effect of temperature could be observed in trial 1 performed on histamine-contaminated tuna stored at three different temperatures. In this case, the highest concentrations of histamine were found in samples stored at 12 °C, which was probably the best condition among those tested for bacterial growth. As shown in the diagram in Figure 2 relating to trial 1, a rapid increase in histamine can be observed as early as 48 h of incubation at 12 and 20 °C, while at 4 °C, the increase has a much slower trend. These data agree with what was reported by Lehane and Olley [34], who observed that HDB growing at refrigeration temperatures usually produce histamine in smaller quantities than species that grow at warmer temperatures, and toxic levels may not be reached. 

Both the postmortem formation of amino acids and their rapid decarboxylation are temperature-dependent. Storage time and temperature are the main factors that affect the production of biogenic amines [63]. Although there is great variability in the results of studies where seafood was kept under different conditions, in general, longer storage times and higher temperatures seem to increase histamine production [64]. However, this statement is not absolute, as it is necessary to consider the bacterial species involved, which may have different optimal development temperatures. For example, among the histaminogenic bacteria often involved, it was found that mesophilic and psychrotolerant variants of *M. morganii* develop under different conditions. The well-known mesophilic variant can produce toxic concentrations of histamine in seafood at or above 7 to 10 °C. In contrast, the psychrotolerant variant can grow and form toxic concentrations of histamine at 2 °C in fresh tuna and at 5 °C in cold-smoked tuna but not at 37 °C [65]. In another study in which canned tuna was inoculated with three different HDB, it was found that at room temperature (20 °C), *M. psychrotolerans* and *M. morganii* produced similar amounts of histamine during the incubation and to a much greater extent than *P. phosphoreum*. Otherwise, at a low temperature (4 °C), *M. psychrotolerans* produced a higher amount of histamine (more than 1000 mg/kg at 8 days) than *P. phosphoreum* (about 556 mg/kg), while no histamine was produced by *M. morganii* [66]. In addition, the possible co-presence of microorganisms capable of producing histaminase should be considered, as they may limit histamine accumulation [34,62,63]. In this case, the histamine concentration may eventually depend on an equilibrium between histamine production and destruction. Trial 2 showed that anaerobic conditions could slow down histamine accumulation in tuna, as can be clearly seen by comparing the diagram of histamine formation in Figure 3 with the diagram of histamine formation at 4 °C under aerobic conditions, displayed graphically in Figure 2. Thus, storage in oil can increase the tuna shelf-life, but this method of food preservation cannot inhibit histamine formation if the fish has been previously contaminated by histaminogenic bacteria. Several studies seem to indicate that the preservation of fish in brine is more effective than preservation in oil in preventing histamine formation. In an investigation carried out on 60 canned fish samples collected from markets in Tehran (Iran), histamine was detected in 46.6% of samples (mean 17.36 ± 15.44, range < LOQ-88 mg/kg). Histamine was not found in samples of tuna in brine (*n* = 9), but it was detected in samples of tuna in oil from the same brand and the same production date (*n* = 18) [67]. Silva et al. [68] investigated histamine levels in 54 samples of canned tuna in Brazil, and 46.3% contained histamine (concentration range of 0.45–83.73 mg/kg). Canned tuna in oil had higher histamine levels compared to tuna in brine for both solid and grated tuna. Moreover, grated tuna contained higher histamine levels compared to solid canned tuna. This could be due to its increased surface area, which could facilitate contamination and enzyme–substrate interactions, as well as longer processing.

In trial 3, in which canned tuna in oil was mixed with vegetables and stored at 4 °C for 8 days, histamine formation did not occur, probably because, in this case, the vegetables did not contain histamine-producing bacteria. Histamine and other BAs can be found in different types of vegetables [31]. In this regard, the above-mentioned study carried out by Silva et al. [68] also included samples of canned tuna in tomato sauce, which showed the highest percent occurrence and levels of histamine. This finding could be due to the contribution of histamine naturally present in tomatoes. A review article on BAs in plant-origin foods reports that significant levels of histamine were recorded in almost all samples of spinach, eggplant, and tomato. This could be explained by the fact that histamine occurs naturally in these foods. Data obtained from different studies report concentration levels in ranges of 4.2–100.6 mg/kg, 9.5–69.7 mg/kg, and 0–17.1 mg/kg in eggplant, spinach, and tomato, respectively [69]. Dala-Paula et al. [70] investigated BAs in some vegetables typical of Brazilian cuisine. Histamine was found in 38.3% of the vegetables, particularly eggplant, jiló, tomato, spinach, parsley, capers, and bean sprouts. The highest concentration levels were detected in eggplant samples (range of 3.69–12.5 mg/100 g, mean of 8.32 mg/100 g). Histamine was also found in commercial vegetable pickles purchased in various retail markets in Van (Turkey), and the concentration levels detected were in the range between 16.54 and 74.91 mg/kg [71]. However, the accumulation of histamine and other BAs in vegetables could also be associated with microbial activity, as occurs in foods of animal origin [69].

For this experiment, canned tuna was used, as it is most commonly used in the preparation of tuna salads. Moreover, according to some authors, canned fish placed on the market is more protected from possible histamine contamination than fresh fish due to stricter quality control standards. In fact, histamine fish poisoning most often occurs after the ingestion of fresh rather than canned fish. However, the canning process itself does not degrade the formed histamine, although the procedure can kill the microorganisms that produce it [72].

Trial 4, performed on a histamine-contaminated tuna steak, showed homogeneous contamination between the outer part and the core of the product, in both cases at rather high concentrations. Such results may be due to the characteristics of the type of fish and the failure to maintain the cold chain after capture, which allowed HDB to proliferate. These bacteria can originate from the marine environment or from post-catching contamination, but most scientists believe that post-harvesting contamination is the main source of histamine formers [34]. Microorganisms are found on all of the outer surfaces (skin and gills) and in the intestines of live and newly caught fish. The fish muscle is sterile at the time of slaughter/capture but quickly becomes contaminated by surface and intestinal bacteria from equipment and humans during handling and processing [73]. 

Trial 5, which involved cooking slices of histamine-contaminated tuna, confirmed that this molecule is resistant to high temperatures, as already reported in the literature [74,75,76]. This means that cooking is not always effective as a preventive measure to reduce the risk of histamine intoxication. In many cases, the subsequent cooking of spoiled fish can only alter the relationship between bacterial numbers and histamine production by reducing or removing the microbial population without significantly affecting the histamine content [34]. A group of Korean researchers evaluated the effect of cooking practices on the concentration of histamine in various foods (seafood, meat, eggs, vegetables, and fermented products). Although there were some differences among the categories of food items, the study’s outcome was that heating processes, such as grilling and frying, increased the histamine levels in foods, while boiling had little influence or even decreased it. Considering that histamine is thermostable, according to the authors, one possible explanation for these changes could be that grilling or frying can cause moisture loss in food, resulting in increased histamine concentrations. Boiling can also result in an increase in histamine concentration, but the effect could be reduced due to dilution. In some cases, the dilution effect caused by water absorption may be prevalent, resulting in a decrease in histamine concentration [74]. 

Trial 6, simulating the use of histamine-contaminated equipment for food handling, demonstrated that non-compliance with hygiene practices and incorrect handling can increase the risk of histamine intoxication. The present experiment also showed the uneven contamination of tuna and salad placed in contact with the contaminated cutting board, probably due to the different distributions of histamine and/or bacterial load on the tool.

**Figure 5:**
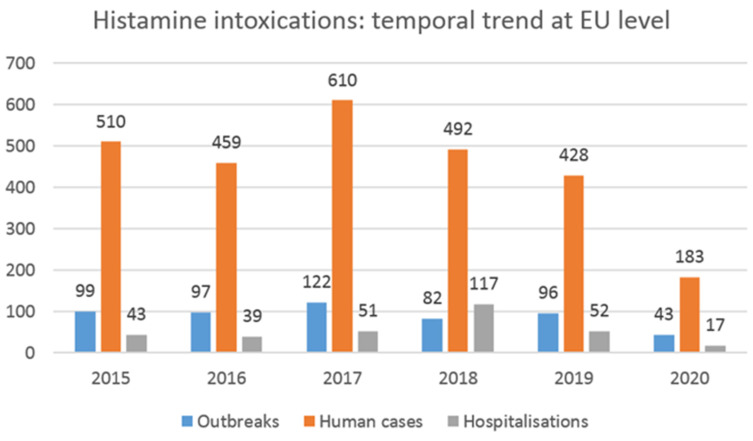
Number of foodborne outbreaks, human cases, and hospitalizations caused by histamine and scombrotoxin in reporting EU member states over the 2015–2020 period (data from EFSA foodborne outbreaks dashboard).

Trial 1 and trial 2 carried out in the present study showed that under certain storage conditions, histamine levels sometimes reached high values, well above the limit established at the EU level for this category of food product (100 mg/kg). Some of these concentrations could give rise to characteristic scombrotoxic fish poisoning symptoms, since it was reported that this intoxication is generally associated with histamine levels above 500 mg/kg in fish [64]. However, there is not a straightforward dose–response relationship, as spoiled fish containing histamine tends to be more toxic than the equivalent amount of pure histamine dosed orally [34]. Bartholomew et al. [77] reported a guideline based on the concentrations of histamine found in fish samples involved in scombrotoxic poisoning incidents. According to the authors, fish with histamine levels below 5 mg/100 g is deemed normal and safe for consumption, while fish with histamine levels between 5 and 20 mg/100 g is considered mishandled and probably toxic. Fish with histamine concentrations between 20 and 100 mg/100 g is classified as unsatisfactory and probably toxic, and finally, fish containing concentrations above 100 mg/100 g is deemed toxic and unsafe for consumption. Recent data collected at the EU level on the number of foodborne outbreaks (FBOs), human cases, hospitalizations, and deaths caused by different agents are available in the European Union’s One Health 2020 report on zoonoses [78]. In the reporting year, the number of outbreaks caused by histamine/scombrotoxin was, in total, 43 (14 strong-evidence and 29 weak-evidence outbreaks), and 183 human cases were reported, of which 17 were hospitalized (9.3% of cases), and 1 died. This is the first-ever reported death from histamine since EFSA began collecting data on outbreaks in 2005. It is possible to observe the trends of these events over time from data extrapolated from the EFSA foodborne outbreaks dashboard [78], as reported in Figure 5.

As can be seen, compared with 2019, the number of FBOs due to histamine poisoning in 2020 decreased considerably (-53 outbreaks, a 55.2% decrease). The temporary closure of all public food service activities, such as restaurants and company and school canteens, as a consequence of the lockdown measures to fight the COVID-19 pandemic clearly impacted the above data [78]. Looking at the data collected by EFSA in the previous years, the highest number of FBOs for histamine poisoning was reported in 2017 (*n* = 122), while no important changes can be observed by comparing the number of FBOs (also human cases) notified in the years 2015, 2016, 2018, and 2019. Considering the five-year period of 2015–2019 (thus excluding the 2020 data, which were affected by the consequences of the pandemic) and comparing it with the previous period of 2010–2014, the total number of registered FBOs due to histamine was 496 and 306, respectively [78,79]. Thus, a considerable increase could be observed in the last five-year period (+190 outbreaks, a 62.0% increase). It will be interesting to analyze the data after 2020 to evaluate whether a significant trend in outbreaks emerges due to this type of intoxication.

## 5. Conclusions

Histamine intoxication is one of the most common diseases caused by fish and fish products. Despite the increasing attention to food safety, the number of cases is increasing, probably due to a change in eating habits and to the increasing consumption of raw fish. Consumers should be made aware of the proper storage of seafood products, from the time of purchase to the time of preparation and the consumption of the products. What emerged in the present study was that improper storage can lead to the formation of histamine at levels far above legal limits, and that storage time and temperature are the main factors that affect this process. While it was to be expected that at 4 °C, histamine production would be slowed, at the other two experimental storage temperatures, it was seen that a lower temperature does not necessarily correspond to less histamine formation. Another interesting finding was that storing tuna in oil can slow the development of histamine, and thus, it could be a method for increasing the shelf-life of this food, whereas cooking does not affect the histamine content once it has formed. The importance of the hygiene of the tools used to handle food should also be emphasized, as they can also be a source of histamine contamination.

The present study focuses on the analysis of histamine in tuna subjected to different conditions. It would be interesting to continue this research in the future from a microbiological perspective to investigate the predominant microflora responsible for the development of this biogenic amine.

## Figures and Tables

**Table 1:** Mass spectrometric parameters for the determination of histamine using an electrospray interface (ESI) in positive ionization mode.

Analyte	MW (g/mol)	Retention Time (min)	Precursor Ion (m/z)	Product Ions (m/z)	CE (eV)
Histamine	111.15	1.75	112.0	68.1	20
				95.2 *	10

* Quantification ion.

**Table 2:** Recovery data for the method for analysis of histamine in tuna samples spiked at 3 concentration levels.

	Histamine Spiking Level (mg/kg)			M ^2^
	20	100	200	
Recovery (%) ^1^	82.9	80.8	78.8	80.8

^1^ Average of 18 replicates for each concentration; ^2^ average recoveries of the 3 spiking levels.

**Table 3:** Results of repeatability and reproducibility tests calculated for analysis of histamine in tuna samples.

Histamine Spiking Level (mg/kg)	Repeatability			Within-Laboratory Reproducibility		
	Mean (mg/kg)	SD ^1^ (mg/kg)	RSD ^2^ (%)	Mean (mg/kg)	SD ^1^ (mg/kg)	RSD ^2^ (%)
20	16.0	1.5	9.6	16.6	1.7	10.4
100	71.0	6.3	8.9	80.8	10.6	13.1
200	148.7	13.1	8.8	157.5	13.9	8.8

^1^ Standard deviation; ^2^ relative SD.

**Table 4:** Histamine concentrations detected in fresh tuna and fresh tuna grafted with tuna muscle naturally incurred with histamine at 6000 mg/kg level after storage at different temperature conditions (trial 1).

		Fresh Tuna	Fresh Tuna Grafted with Tuna Muscle Incurred with Histamine
Day	Temperature (°C)	Histamine (mg/kg)	Histamine (mg/kg)
	4	<LOD	12.8
1	12	<LOD	23.9
	20	<LOD	12.0
	4	<LOD	33.2
2	12	<LOD	50.5
	20	<LOD	31.3
	4	<LOD	36.7
3	12	<LOD	119.9
	20	<LOD	92.1
	4	<LOD	24.8
4	12	<LOD	1228.9
	20	<LOD	919.2
	4	<LOD	45.0
5	12	<LOD	2247.0
	20	<LOD	1681.0
	4	<LOD	68.2
6	12	<LOD	2721.3
	20	<LOD	N.D. ^1^

^1^ Not determined.

**Table 5:** Histamine concentrations detected in fresh tuna and tuna salad placed in contact with the cutting board previously used to cut tuna muscle naturally incurred with histamine at 8000 mg/kg (trial 6).

Samples	Histamine (mg/kg)
Fresh tuna—slice A	59.3
Fresh tuna—slice B	125.3
Fresh tuna—slice C	95.4
Fresh tuna—slice D	535.9
Tuna salad	240.7

## Data Availability

The data presented in this study are available in the article.
